# Ejaculation-Triggered Reversible Cerebral Vasoconstriction Syndrome: A Case Report with Longitudinal Neuroimaging

**DOI:** 10.3390/diagnostics16152360

**Published:** 2026-07-27

**Authors:** Koji Hayashi, Yoshihiro Ito, Mamiko Sato, Yuka Nakaya, Toshiaki Takehara, Asuka Suzuki, Toyoaki Miura, Kouji Hayashi, Yasutaka Kobayashi

**Affiliations:** 1 Department of Rehabilitation Medicine, Fukui General Hospital, Fukui 910-8561, Japansatomoko@f-gh.jp (M.S.);; 2 Graduate School of Health Science, Fukui Health Science University, Fukui 910-3190, Japan

**Keywords:** reversible cerebral vasoconstriction syndrome, thunderclap headache, vasoconstriction, coitus, ejaculation, headache disorders, primary

## Abstract

A 40-year-old man presented with thunderclap headache (TCH) and vomiting post-masturbatory ejaculation. He reported a similar episode with posterior neck pain five days earlier after ejaculation. Unlike his migraine history, these headaches were unusually severe. Upon arrival, physical and neurological examinations were unremarkable except for an elevated blood pressure (154/110 mmHg). Brain MRI/MRA systematically ruled out intracerebral/subarachnoid hemorrhages, aneurysms, cerebral venous thrombosis, and cervical artery dissection, with preserved flow voids on B-PASS, T2, and T2-FLAIR. Notably, MRA demonstrated multiple segmental vasoconstrictions compared to a baseline MRA from five years prior. With an RCVS_2_ score of 8, he was diagnosed with reversible cerebral vasoconstriction syndrome (RCVS). Symptoms resolved within 10 days of starting oral verapamil (120 mg/day), and follow-up MRA at one month showed partial improvement. This case highlights RCVS manifesting as a secondary cause sharing features with the proposed headaches associated with sexual activity (HSA) spectrum. RCVS is the predominant secondary cause of HSA (67–90%), often indistinguishable from primary HSA at onset due to the shared presentation of sudden TCH. Its pathophysiology likely involves an orgasm-induced sympathetic surge causing transient dysregulation of cerebral vascular tone. This case emphasizes that thorough neurovascular imaging is important in HSA to differentiate RCVS from primary headaches, ensuring the prompt initiation of calcium channel blockers and the avoidance of triptans.

**Figure 1:**
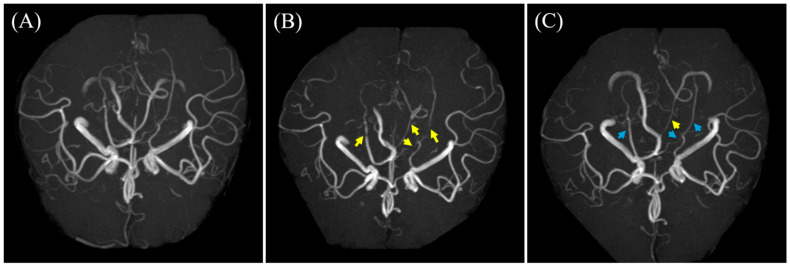
**Magnetic resonance angiography (MRA) findings.** (**A**) MRA performed five years prior to the current onset (at age 35), showing normal vessel diameters. (**B**) MRA at onset, demonstrating multiple segmental vasoconstrictions in the posterior circulation, including the bilateral posterior cerebral arteries and left vertebral artery (yellow arrows). (**C**) MRA one month after onset, showing partial resolution of the previous vasoconstrictions, including the bilateral posterior cerebral arteries (blue arrows), although some segments show no significant improvement, including the left vertebral artery (yellow arrow). All three MRA examinations were performed using a 1.5-Tesla scanner (GE Healthcare, Chicago, IL, USA) with a three-dimensional time-of-flight (3D-TOF) sequence. He had a roughly 25-year history of migraine without aura, occurring approximately 4–5 times per month. His typical migraine attacks were characterized by unilateral, throbbing pain, usually lasting for 12 to 48 h, and were associated with nausea, photophobia, and phonophobia. These attacks typically responded well to NSAIDs or triptans. Notably, the baseline MRA at age 35 was performed when he visited our hospital for the evaluation of a typical migraine attack, which confirmed completely normal vessel diameters at that time. In contrast, the presenting thunderclap headaches (TCHs) were distinctly different from his usual migraine episodes. While his migraines had a gradual onset, the TCHs reached maximal intensity within seconds immediately following ejaculation. Furthermore, the intensity of the TCHs was unprecedented and excruciating (VAS 10/10), and they were accompanied by vomiting, which was not a typical feature of his previous migraine attacks. The patient reported no recent use of illicit substances, such as cannabis or cocaine, nor any exposure to vasoactive medications, including triptans or over-the-counter nasal decongestants, prior to the current onset. Reversible cerebral vasoconstriction syndrome (RCVS) is a clinical–radiological syndrome characterized by abrupt, severe headaches and multifocal cerebral arterial constriction that typically resolves within three months [1,2]. Therefore, the partial improvement observed on MRI at the one-month mark in our case is consistent with previously reported clinical courses. While triggers include vasoactive substances and the postpartum period, sexual activity is a well-established precipitant, accounting for approximately 10.5% of cases [3]. The present case of a 40-year-old man illustrates RCVS manifesting as a form of headache associated with sexual activity (HSA), as his TCH was specifically triggered by ejaculation [4,5]. Additionally, the RCVS_2_ score proposed by Rocha et al. is used for diagnosing RCVS; a score of ≥5 has been reported to have 99% specificity and 90% sensitivity [6]. The patient in this case had a score of 8 points (recurrent TCHs: 5, vasoconstrictive trigger: 3, no carotid involvement: 0, male sex: 0, no SAH: 0). Distinguishing between primary HSA and RCVS is a critical diagnostic challenge [4]. Primary HSA is classified as a primary headache disorder occurring only during sexual activity in the absence of any intracranial disorder [4]. Conversely, RCVS is a secondary cause defined by the presence of reversible segmental vasoconstriction [1,2]. Despite these classifications, the two entities are clinically indistinguishable at onset, as both frequently present as sudden, explosive TCHs [4]. In the present case, the availability of previous MRA facilitated a prompt diagnosis; however, in emergency settings where prior imaging is unavailable for comparison, the clinical distinction between HSA and RCVS becomes increasingly challenging. Research has proposed a relationship between these entities, suggesting they might exist on a shared pathophysiological spectrum driven by an orgasm-induced sympathetic surge that elevates catecholamines and precipitates transient dysregulation of cerebral vascular tone [5]. Notably, among patients presenting with headaches triggered by sexual activity, within HSA spectrum, RCVS is the predominant secondary cause, accounting for 67% to 90% of such cases [4,5]. RCVS can cause ischemic or hemorrhagic strokes, and its management differs fundamentally from primary headaches [2]. Although a history of migraines is frequently associated with RCVS, distinguishing RCVS from typical attacks is crucial; avoiding triptans—which can induce or exacerbate vasoconstriction—and initiating calcium channel blockers should be considered to prevent neurovascular complications [1,2]. Given that RCVS is the predominant secondary cause of HSA, exhaustive neurovascular imaging via MRI and MRA is important in the diagnostic evaluation to exclude life-threatening conditions and ensure appropriate therapy. As a limitation, we were unable to obtain follow-up MRI data from three months or more post-procedure to confirm the complete recovery of the vessel diameter to its baseline level from five years prior. Having access to these follow-up images would have significantly strengthened our findings and provided a more compelling presentation of the long-term outcomes.

## Data Availability

The original contributions presented in this study are included in the article. Further inquiries can be directed to the corresponding author.
